# Examining household effects on individual Twitter adoption: A multilevel analysis based on U.K. household survey data

**DOI:** 10.1371/journal.pone.0297036

**Published:** 2024-01-25

**Authors:** Shujun Liu, Luke Sloan, Tarek Al Baghal, Matthew Williams, Paulo Serôdio, Curtis Jessop

**Affiliations:** 1 School of Social Sciences, Cardiff University, Cardiff, The United Kingdom; 2 Institute of Social and Economic Research, University of Essex, Colchester, The United Kingdom; 3 National Centre for Social Research, London, The United Kingdom; Federal University of Goias: Universidade Federal de Goias, BRAZIL

## Abstract

Previous studies mainly focused on individual-level factors that influence the adoption and usage of mobile technology and social networking sites, with little emphasis paid to the influences of household situations. Using multilevel modelling approach, this study merges household- (*n*_*1*_ = 1,455) and individual-level (*n*_*2*_ = 2,570) data in the U.K. context to investigate (a) whether a household economic capital (HEC) can affect its members’ Twitter adoption, (b) whether the influences are mediated by the member’s activity variety and self-reported efficacy with mobile technology, and (c) whether the members’ traits, including educational level, gross income and residential area, moderate the relationship between HEC and Twitter adoption. Significant direct and indirect associations were discovered between HEC and its members’ Twitter adoption. The educational level and gross income of household members moderated the influence of HEC on individuals’ Twitter adoption.

## Introduction

The widespread availability of mobile technology has made it increasingly common for people to use social networking sites in their personal and professional lives [1]. These social networking sites, such as Twitter (also named as X.), enable users to communicate text updates with their network, serving not only as a tool for expressing personal opinions and thoughts, but also as a “digital agora” where individuals can engage with one another [2]. The interaction process in turn provides individuals potential resources and information that might result in more tangible benefits [3].

Numerous studies have sought to investigate the factors that influence an individual’s adoption of hardware (e.g., mobile phone) and software mobile technologies (e.g., Twitter). However, existing research mostly focused on individual-level antecedents, such as demographic characteristics and psychological motivations [4–6]. Fewer studies explored the role of household factors. As people’s traits, perceptions and behaviors are rooted in experiences in their families of origin [7], it is likely that the household elements may affect their attitudes and actions toward social networking sites as well [8]. Among a series of household factors, household economic capital (HEC) is especially critical, as other resources, such as social and cultural capital, are often derived from it [9].

To better comprehend how household factors affect an individual’s usage of mobile technology and social networking sites. This study focuses on their influence on mobile technology usage and Twitter adoption. Twitter is the most popular microblogging site enabling users to share brief, real-time messages and interact through likes, comments, and retweets. It has been widely used around the world with currently 330 million users [10]. Specifically, we merge data from both household and individual levels using multilevel modelling approach (MLM), seeking: (a) to investigate the effect of HEC on an individual’s Twitter adoption, (b) to examine how an individual’s *activity variety* and *self-reported efficacy* with mobile technology mediate the association between HEC and Twitter adoption and (c) to explore potential moderating effects of an individual’s educational level, personal income and residential area, on the association between HEC and his/her Twitter adoption.

This study is situated in the context of United Kingdom. As of 2022, there were 57.6 million active social media users in the U.K., representing a social media penetration rate of 84.3 percent of the population [11]. Also, the use of mobile devices has become increasingly popular in the U.K. over the past decade, with six in ten internet users citing smartphones as their primary device for accessing the internet in 2020 [12]. The thriving markets of social networking sites and mobile technology make the U.K. an ideal setting for this study.

## Literature review

### Household economic capital and individual use of mobile technology

The diffusion of innovation (DOI) theory is often used to comprehend the factors that influence the attitudes and behaviors toward mobile technology and social networking sites. The theory describes “the process in which an innovation is communicated through certain channels over time among the members of social system.” [13]. Scholars studying the process of diffusion often associate the adoption of new ideas, practices or technologies with a specific communication channel, a social structure or a given system of values or culture to comprehend the potential mechanism that facilitates usage and adoption [14]. Among a range of adoption behaviors, a strand of DOI research in particular focuses on the adoption or usage of information and communication technologies (ICTs) [15], such as cable television [16], personal computer [17], mobile phones [18, 19] and social media sites [20].

Previous studies suggested that the adoption or usage of ICTs are subject to a wide range of factors, which can be identified at the individual-, organizational- and national-levels. At the individual level, scholars identified sociodemographic traits, such as higher income, better education, and younger age as key predictors of adoption behavior [6, 21], essentially corresponding to Rogers’s description of “innovators” [13]. There were also some studies examining the role of psychological traits or needs in determining the innovation adoption [22, 23]. At the organizational- and the national-levels, scholars placed greater emphasis on characteristics of the organization or the country itself [24, 25]. For instance, some scholars discovered that countries with higher research and development (R&D) expenditure, human resources and innovation output are more likely to adopt innovative technologies [26].

Although scholars have agreed on some factors affecting usage of ICTs at various levels, the influence of households, specifically household capital, has not been adequately addressed. Capital can be defined as the accumulated labor in material or embodied form that enables individuals or groups to harness social energy in the form of reified or living labor [27]. Accordingly, household capital refers to the household acquisition of material and symbolic goods that can either support or degrade household members. Among a series of household capital indicators (i.e., economic, social, cultural, and symbolic), economic capital, commonly referring to household incomes and assets that can be readily converted into cash [28], typically takes the lead to determine the possession of other capitals [29]. Studies on adoption of ICTs by individuals and organizations (e.g., firm) have indicated that economic capital plays a crucial role in determining attitudes or behaviors toward innovative technologies. Individuals and organizations with greater economic resources are better equipped to invest in and implement new technologies to enhance their lives or grow their business [6, 30]. Regarding this, it is possible that variations in the economic capital of households may also contribute to its members’ different behaviors and perceptions toward innovative technology.

**Fig 1:**
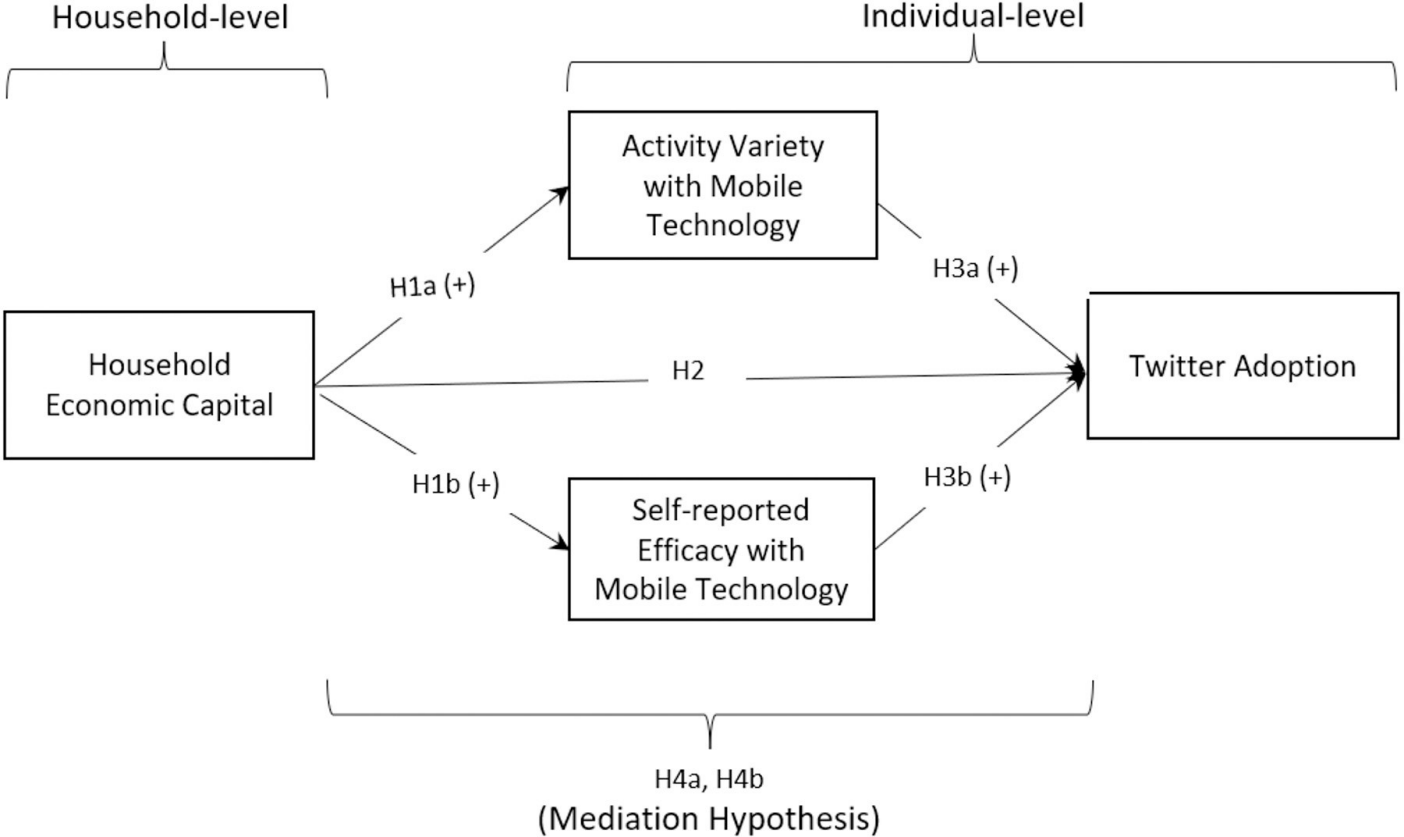
Summary of hypotheses and proposed model.

In line with this, we first investigate the impact of household economic capital on its members’ behaviors and attitudes toward mobile technology, specifically focusing on activity variety and self-reported efficacy with mobile technology. Activity variety with mobile technology refers to the range of different tasks and experiences that individuals can engage in with mobile devices [31]. Self-reported efficacy with mobile technology refer to an individual’s perception of their ability to effectively and efficiently use mobile devices to accomplish tasks and achieve desired outcomes [32]. Individuals coming from households with greater economic capital may possess more resources and opportunities to develop technology capital, including access to technology devices and high-speed internet, as well as owning technology-related knowledge and skills. Consequently, they may participate in a broader array of activities and exhibit greater ease in using mobile technologies. Accordingly, the following hypothesis is proposed (see Fig 1):

**H1:** Economic capital of a household will be positively associated with its members’ (a) activity variety and (b) self-reported efficacy with mobile technology.

In addition to the behaviors and attitudes toward hardware mobile technology, the economic capital of a household may also affect its members’ adoption of software applications, such as Twitter. According to the Pew Research Center, the U.S. citizens from higher-income households were more likely to use social networking sites [33]. This trend may also apply to the U.K. context. Therefore, the following hypothesis is proposed:

**H2:** Economic capital of a household will be positively associated with its members’ adoption of Twitter.

### Individual use of mobile technology and Twitter adoption

Apart from household-level influences, there could be an association between individuals’ behaviors and attitudes toward mobile technology and their adoptions of social network sites. The widespread availability of mobile technology has led to the emergence of a new phenomenon called “networked individualism,” where individuals are empowered by the ability to maintain constant connections with diverse social network applications [34]. In line with this, Lin and Lu (2011) [35] argued that the desire to engage in social networking is a key motivating factor driving the usage of mobile technology. Put differently, it is likely that individuals who have greater knowledge and engage in more types of activities with mobile technology are more inclined to use social network sites. Previous empirical research also supports this proposition. For instance, Hargittai and Litt [36] discovered that the variety of internet activities a user has could affect their decisions to adopt Twitter. Accordingly, the following hypothesis is proposed:

**H3:** Individuals’ (a) activity variety and (b) self-reported efficacy with mobile technology will be associated with their adoptions of Twitter.

Based on H1 and H3, economic capital of a household will drive its members’ activity variety and self-reported efficacy with mobile technology, which, in turn, will be associated with their adoptions of Twitter. Put differently, an individual’s activity variety and self-reported efficacy with mobile devices will mediate the association between economic capital at the household level and Twitter adoption at the individual level. Therefore, we put forward that:

**H4:** The effect of household economic capital on its members’ Twitter adoptions will be mediated by their (a) activity variety and (b) self-reported efficacy with mobile technology.

### Potential moderators at individual level

In addition to household-level factors, individual traits may also play a role in adoption of social networking sites. For instance, Bobkowski and Smith [37] discovered that social media users tend to have higher levels of economic stability, education, and perceived social support. Feng et al.’s [6] meta-analysis on the sociodemographic factors affecting social media adoption revealed that individuals who are female, younger, well-educated, well-paid and urban residents are more likely to use social networking sites. The findings revealed by the Pew Research Center [33] further confirmed that social media users are predominantly younger, female and have higher levels of education and income. It can be seen from these findings that people owning more resources (e.g., financial, intellectual et al.) are more likely to use social networking sites. The reason may be that individuals can transfer their belongings from one field to another, and offline practices and habits, therefore, are more likely to reappear online [38].

#### Educational level

Among the multiple influential factors, some scholars agreed that people with higher educational levels are more likely to use social networking sites. Others conclude otherwise, arguing that people with lower educational levels typically have less social capital, and they have to turn to cyberspace for more interactions [39, 40]. Given the mixed findings, it is possible that educational level and household economic capital jointly influence actions toward social networking sites. In other words, a person’s educational level may moderate the relationship between HEC and Twitter adoption. Furthermore, compared to people with higher levels of education, household conditions may compensate for or support people with lower education. Thus, the association between HEC and Twitter adoption might be stronger among those with lower-level education. In line with this, the following hypothesis is proposed.

**H5:** Individuals’ educational level will moderate the association between their household economic capital and their Twitter adoptions. The association will be stronger for those with lower educational levels compared to those with medium and higher educational levels.

#### Individual income

The same logic may be true for the factor of individual income. Although most prior studies found that personal income affects social media use positively [6, 20], it is likely that household income might moderate this association. Specifically, for lower-income individuals, the increase on household economic capital would provide relatively more resources compared to that for higher-income individuals. As household and individual income might collectively determine a person’s behavior on social networking sites, the following hypothesis is proposed:

**H6:** Individuals’ income will moderate the association between their household economic capital and their Twitter adoptions. The association will be stronger for those with less individual income compared to those with medium and higher individual income.

#### Residential area

Finally, it is possible that the association between household economic capital and individuals’ Twitter adoptions may be stronger for those residing in the rural areas. The reason is that limited access to the internet and technology in rural areas might hinder the usage of social networking sites, making affluent rural household comparatively better equipped than their urban counterparts to provide members with necessary resources. Conversely, technology innovations are more widely disseminated in urban areas, resulting in a weaker effect of household economic conditions on individuals’ Twitter adoption [41, 42]. As such, we put forward the following hypothesis:

**H7:** Individuals’ residential area will moderate the association between their household economic capital and their Twitter adoptions. The association will be stronger for rural residents compared to urban residents.

## Method

### Data and sample

Data were derived from the UK Understanding Society Innovation Panel (IP), part of the UK Household Longitudinal Study (UKHLS), an annual panel survey of UK households. The IP survey is a longitudinal sample of individuals within the household context. Thus, the core structure of the IP survey consisted of (a) a household questionnaire and (b) an adult individual interview and self-completion questionnaire (aged 16+) [43].

The first wave of the IP survey was carried out in 2008 [44], targeting households from England, Scotland and Wales with a stratified and geographically cluster design. First, a systematic random sample of sectors was drawn with probability proportional to population size. Then, within each selected sector, a certain number of addresses were chosen by systematic random sampling approach, resulting in a total sample of addresses. Finally, for each sampled address, the interviewer identified the sampled persons. 2,760 addresses were sampled in the first wave. Only the responding households of previous wave were paid a visit again in the subsequent wave. An additional 960, 1,560 and 960 new addresses were added as the refreshment sample in waves 4, 7 and 10 [43].

This study uses the dataset of Wave 10, which was collected in May 2017 and included a series of questions regarding social media usage. The issued sample at Wave 10 consisted of 1,455 households. Correspondingly, 2,570 individuals from these households were interviewed. To examine the variance at the household- and individual-level simultaneously, this study merged the household- and individual-level datasets [45].

### Variable constructions

#### Household-level

*Household economic capital*. The item about family gross monthly incomes from Wave 10 was used as a proxy of household economic capital. This item was derived by summing up the values of total income of all residents in the month before the interview in the household.

#### Individual-level

*Variety of activities in mobile technology*. 26 items, of which half were used to survey participants’ activities on smartphone and other half for examining activities on tablet, were used to measure activity variety on mobile technology (e.g., “Do you use your smartphone for browsing websites,” “Do you use your smartphone for playing games”). Each item was dummy coded (1 = *Yes*, 0 = *No*). These items were summed up and averaged to produce an index, ranging from 0 (*Adopt none*) and 1 (*Adopt all of them*).

*Self-reported efficacy on mobile technology*. Two items were used to assess participants’ self-reported skill level with mobile technology (e.g., “how would you rate your skills of using a smartphone/tablet”). The answers range from 1 (*Beginner*) to 5 (*Advanced*). The two items were summed up and averaged, ranging from 0 (*No skills at all*) to 5 (*Advanced*).

*Twitter adoption*. Twitter adoption was measured using the binary item “Do you have a personal Twitter account?”. 1 was given if the answer was yes, otherwise 0 was recorded.

*Moderators*. *Educational level* is measured using the item regarding the highest level of education that participants have attained. This item was reverse-coded ranging from 1 (*No qualification*) to 5 (*Doctorate degree*). The option of “other qualification” was coded as 0, serving as the reference category. *Individual income* is calculated by summing up a person’s monthly earnings. *Residential area* is a dichotomous variable with 1 representing *urban area* and 0 representing *rural area*.

*Covariates*. Gender (coded as 1 = *Male* and 0 = *Female*), relationship status (coded as 1 = *Living with partnership* and 0 = *Living alone*) and age were included in all multilevel mediation models.

### Analysis procedure

Analysis of missing data was first conducted using multiple imputation approach. The data was then standardized for variables such as household economic capital, personal income and age to avoid the effects of large variance. Following, we checked the absolute skewness and kurtosis values of each variable to assess if they are normally distribution. The results showed that the values of all variables are between -2 and 2, except for HEC and personal income. Using box plot [46], we identified the outliers of these two variables and replaced them with the 99^th^ percentile for these two variables. Table 1 shows the descriptive statistics for all variables.

**Table 1:** Descriptive statistics and bivariate correlation.

	M	SD	Range	1	2	3	4	5	6	7	8	9	10
**Twitter**	.21	.41	0–1	1									
**HEC**	-.02	.96	-2.66–3.76	.21***	1								
**Activity**	.36	.27	0–1	.37***	.27***	1							
**Efficacy**	2.28	1.65	0–5	.36***	.26***	.87***	1						
**Residential**	.76	.43	0–1	.01	-.07***	.01	.03	1					
**Income**	-.02	.88	-1.86–3.92	.09***	.53***	.19***	.18***	-.07***	1				
**Education**	3.03	1.55	0–5	.18***	.30***	.27***	.28***	-.05*	.33***	1			
**Gender**	.47	.59	0–1	-.34***	.02	-.04	-.002	-.02	.22***	.01	1		
**Relationship**	.51	.50	0–1	-.05	.20***	-.04*	-.03	-.11***	.16***	.11***	.04*	1	
**Age**	.01	1.00	-1.85–2.50	-.34	-.23***	-.49***	-.49***	-.13***	.05**	-.16***	.02	.31***	1

Twitter = Twitter adoption, HEC = household economic capital, Activity = activity variety on mobile technology, Efficacy = self-reported efficacy on mobile technology, Residential = residential area, Income = individual income, Gender, relationship and age are control variables.

*p < .05

**p < .01

***p < .001

To test the association among the dependent variable, mediators and independent variable, three steps of mediation analysis based on Baron and Kenny [47] were used, including (step1) the direct effect of independent variable on dependent variable, (step2) the direct effect of independent variable on mediating variable, and (step 3) the indirect effect of independent variable on dependent variable, controlling mediators. After that, the causal mediations were examined using package of mediation in R.

### Model selection

Because individuals are clustered within households, individuals’ Twitter adoption may be correlated within households, and different households may have different random effects on one’s Twitter adoption posed by HEC. This study used multilevel modeling (MLM) (also known as mixed-effect model) with the package of *lme4* in *R* to take such heterogeneity within and between households into consideration. Multilevel modelling comprises both fixed effects and random effects. The former assumes that the explanatory variable has a fixed or constant relationship with the response variable across all observations; while the latter assumes the fixed effects may vary across different groups. That is, the estimates of the fixed effects are conditional on the random effects, which are represented by random intercept and slope [48].

To avoid the over-fit of the modelling, we only kept the random intercept for each household, which represents the intercept deviation of the household from the global intercept. For analyses with continuous outcome (i.e., activity variety and self-reported efficacy), linear mixed model (LMM) was used; while for the binary outcome (i.e., Twitter adoption), generalized linear mixed model (GLMM) was employed. To better interpret coefficients of logistic regression (i.e., log-odds), we calculate the average marginal effects for independent variables (presented in parentheses).

## Results

Before conducting the mediation and moderation analyses, the bivariate correlations were calculated among the variables (see Table 1). Most of the predictors, including household- and individual-level, were significantly correlated with Twitter adoption. In particular, there were significant positive correlations between activity variety and Twitter adoption (*r* = .37, *p* < .001), as well as between self-reported efficacy and Twitter adoption (*r* = .36, *p* < .001), providing supports for H3a and H3b.

To check the proportion of variance explained by the random effect, we looked at the intraclass correlation coefficient (ICC; ICC = between-group variance/between-group variance + within-group variance) of the null model (unconditional model) (see Model 0 in Table 2). Result showed ICC is .22, meaning that 22% of the variance can be explained by the household structure in the observations. This also provides evidence of the necessity to use a multilevel modeling approach.

**Table 2:** Results of multilevel mediation analysis.

	Model 0	Model 1	Model 2a		Model 2b		Model 3
	Twitter adoption	Twitter adoption	Activity on Mobil Tech		Efficacy on Mobil Tech		Twitter adoption
	*β (SE)*	*β (SE)*	*β (SE)*	*t*	*β (SE)*	*t*	*β (SE)*
**Fixed effects**							
**Step 0**							
** Constant**	-1.64 (0.09)***						
**Step 1**							
** Constant**		-2.13 (.14)***					
** HEC**		.40 (.07)***					
**Step 2**							
** Constant**			.35 (.01)***	41.91	2.13 (.05)***	42.87	
** HEC**			.04 (.01)***	7.38	.22 (.04)***	6.18	
**Step 3**							
** Constant**							-3.42 (.22)***
** HEC**							.30 (.07)***
** Activity**							1.97 (.40)***
** Efficacy**							.22 (.07)**
**Control**							
** Age**		-1.14 (.09)***	-.13 (.01)***	-25.03	-.81 (.03)***	-25.80	-.81 (.09)***
** Gender**		.09 (.12)	-.02 (.01)*	-2.01	.02 (.05)	.34	.13 (.12)
** Relationship**		.41 (.14)**	.05 (.01)***	4.40	.36 (.06)***	5.70	.24 (.15)
**Random effects**							
** Household variance**	.92 (.96)	.76 (.87)	.02 (.12)		.55 (.74)		.68 (.82)
** Residual variance**			.04 (.20)		1.42 (1.19)		
**ICC**	.22	.19	.28		.28		.17

For Model 0, 1 and 3, coefficients are log-odds. Generalized linear mixed models (Model 0,1 and 3) were fit by maximum likelihood. Linear mixed models (Model 2a and 2b) were fit by restricted maximum likelihood. LMM models in *R* return the *t-value*. To better comprehend the results, *p-values* of LMM were computed based on conditional *F*-test

### Multilevel mediation analysis

The results of Step1 indicated (see Model 1 in Table 2), the total effect of HEC on the Twitter adoption of household’s members was significant (*b =* .40, 5.06%, *SE* = .07, *p* < .001), implying that if one’s household income increases one unit, their probability to adopt Twitter improve by around 5%, providing supports for H2.

In Step 2, we investigated the association between HEC and individual usage of mobile technology (Model 2a and 2b in Table 2). The finding showed that HEC was significantly associated with the household member’s activity variety (*b* = .04, *SE* = .01, *p* < .001) and self-reported efficacy with mobile technology (*b* = .22, *SE* = .04, *p* < .001). Therefore, H1a and H1b were supported.

Step 3 looked at the indirect effect of HEC on Twitter adoption while controlling for the two mediators. Results of Model 3 (see Table 2) revealed that the effect of HEC on Twitter adoption was still significant (*b* = .30, 3.67%, *SE* = .07, *p* < .001), but to a lesser extent than Model 1. This suggested that the effect of HEC on Twitter adoption was mediated by one’s activity variety and self-reported ease regarding mobile technology.

At last, we conducted two mediation analyses respectively with activity variety and self-reported efficacy with mobile technology to examine if the indirect effects are significant. Based on *quasi-Bayesian Monte Carlo* method with 100 simulations, the indirect effect of HEC on the individuals’ Twitter adoption was found to be significant via their activity variety (*b* = .01, *95% CI* = .01 –.02) (see Table 3). This indicated that of the estimated increase in probability of adopting Twitter (i.e., 4.90%, *95% CI* = .03 –.07) because of the increase in HEC, an estimated 1.1% was due to the activity on mobile technology and remaining 3.79% (*95% CI* = .02 –.05) was from HEC itself. The mediator of activity variety on mobile technology accounts for approximately 22.02% of the total effect [P_indirect_ = (.011) / (.049)].

**Table 3:** Direct and indirect effects summary.

Relationship	Total effect	Direct effect	Indirect effect	Conclusion
**HEC → Activity → Twitter Adoption**	.049	.038	.011 (22.02%)	Partial mediation
**HEC → Efficacy → Twitter Adoption**	.044	.038	.006 (13.24%)	Partial mediation

Cell entries are the increase the probability of adopting Twitter through direct, indirect, and total effects. Total effect is the combination between (1) direct effect from HEC to Twitter adoption and (2) the indirect effects of HEC on Twitter adoption respectively flowing through the two mediators.

Similarly, HEC was found to indirectly affect one’s Twitter adoption via self-reported efficacy on mobile technology (*b* = .006, *95% CI* = .002 –.01). That is, of the estimated increase in the probability of adopting Twitter (4.40%, *95% CI* = .03 –.06) due to the HEC increase, an estimated 0.60% was as result of the self-reported efficacy and the remaining 3.80% (*95% CI* = .02 –.05) was because of HEC itself. The mediator of self-reported efficacy on mobile technology accounts for around 13.24% of the total effect [P_indirect_ = (.006) / (.044)].

### Multilevel moderation analysis

Moderation hypotheses were assessed using package of *interactions* in *R*. Each of the three moderators (i.e., individual education, income and residential area) was tested for the path from HEC to Twitter adoption. The significance of interaction item between HEC and moderators was first examined. If the interaction term is significant, we proceeded with slope analysis. Specifically, we calculated the slopes of the relationship between HEC and Twitter adoption at the mean and one SD above and below the moderator mean [49].

**Fig 2:**
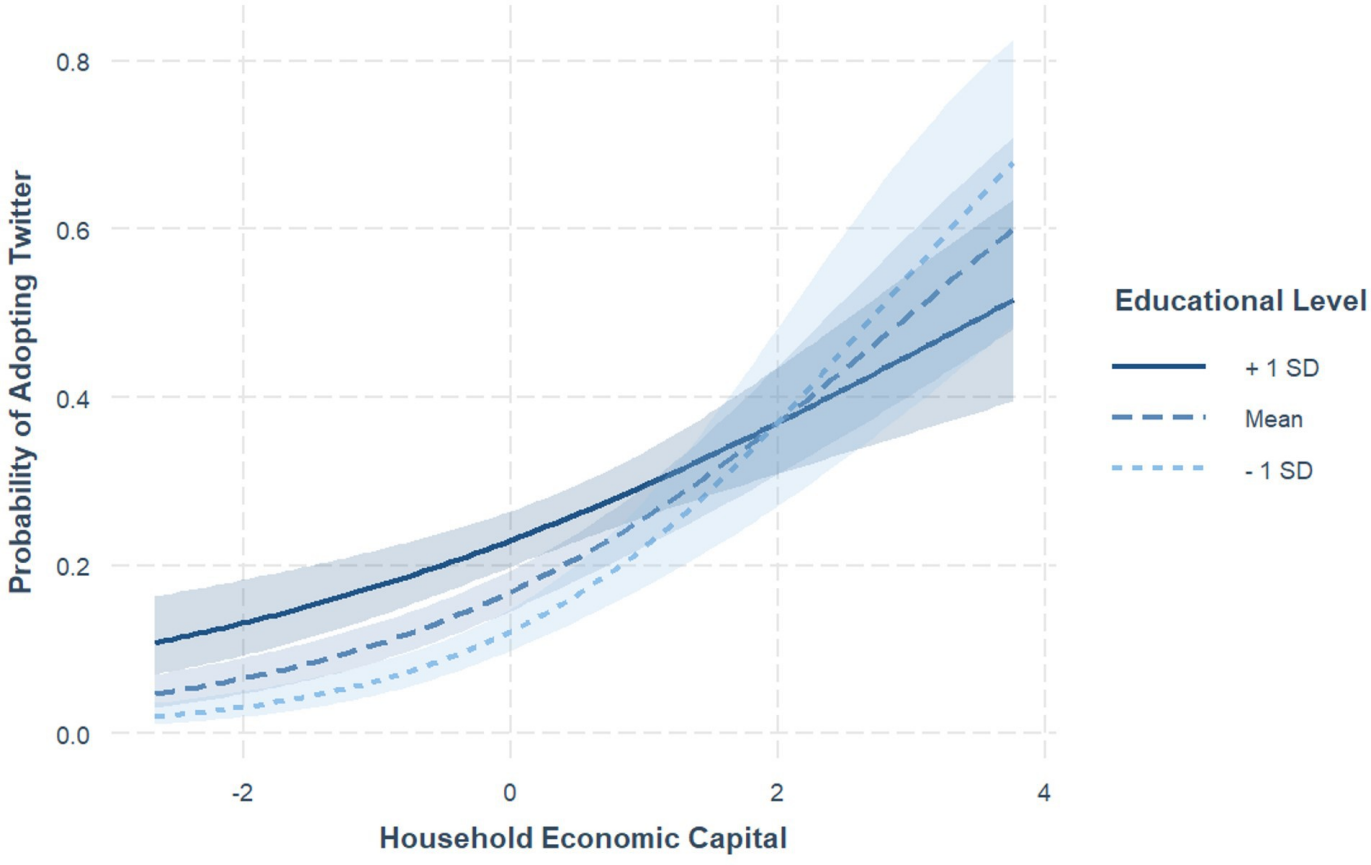
Individual educational level as moderator on the association between HEC and Twitter adoption.

The results of the moderation model showed that a person’s educational level negatively moderated the relationship between HEC and one’s Twitter adoption (*b* = -.13, *SE* = .04, *p* = .002), demonstrating a notable variation in the relationship between HEC and Twitter adoption among individuals with different education levels (see Fig 2). Specifically, this association was more pronounced in individuals with lower education levels, as indicated by a steeper slope (*b* = .73, *SE* = .11, *p* < .001). In contrast, for those with medium and higher education levels, the relationship was comparatively weaker, as reflected by less steep slopes (medium education: *b* = .53, *SE* = .07, *p* < .001; higher education: *b* = .34, *SE* = .07, *p* < .001). This suggests that the influence of HEC on Twitter adoption is significantly moderated by the individual’s level of education, providing support for H5.

**Fig 3:**
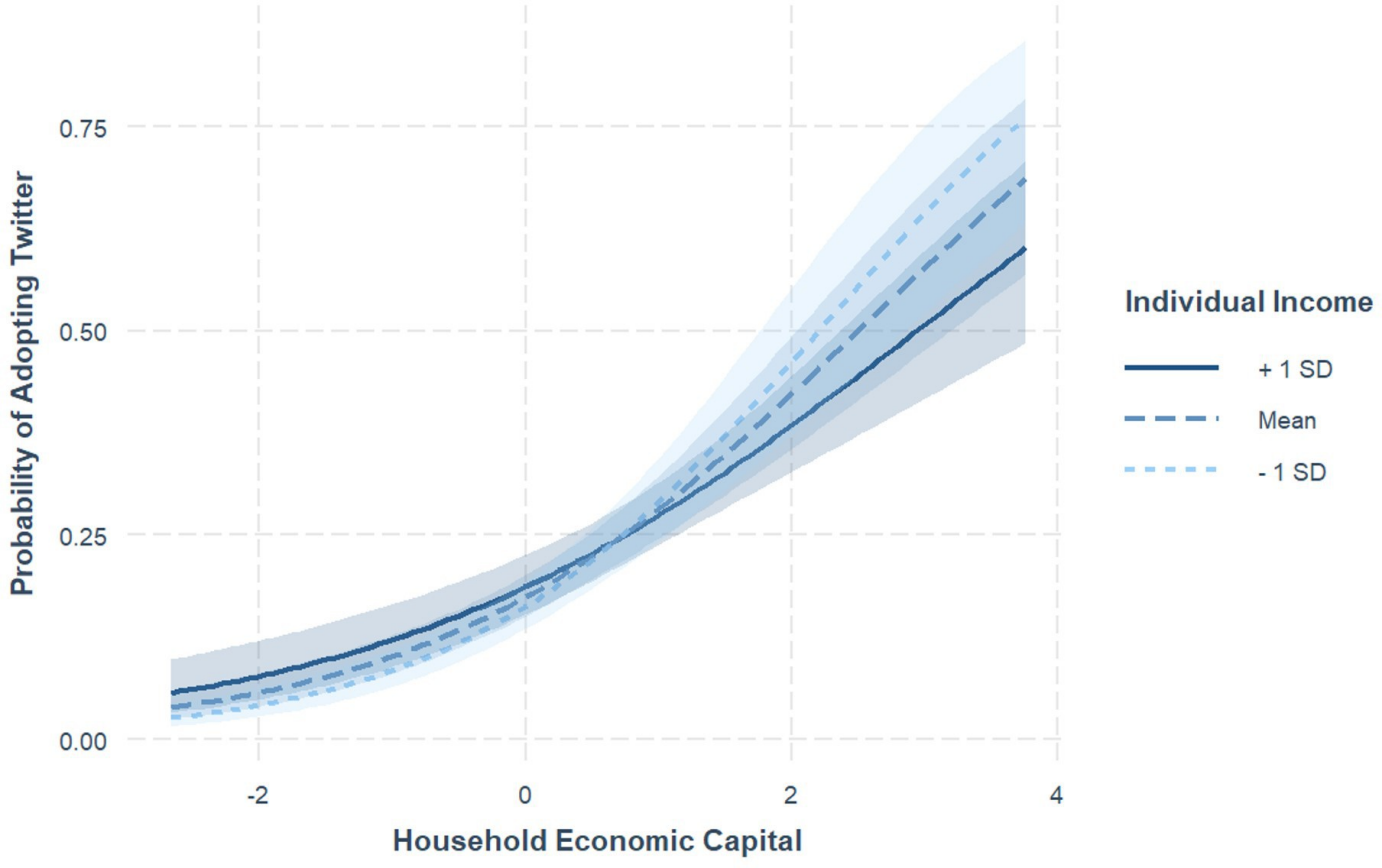
Individual income as moderator on the association between HEC and Twitter adoption.

The analysis of H6, which examined the moderating effect of individual income on the relationship between HEC and Twitter adoption, revealed a significant interaction (*b* = -.14, *SE* = .04, *p* = .001). This means that this interaction was characterized by varying strengths of association across different income levels (see Fig 3). For individuals with lower incomes, the influence of HEC on Twitter adoption was notably stronger (*b* = .74, *SE* = .08, *p* < .001), as indicated by a steeper slope. In comparison, this association was less pronounced for those with medium (*b* = .62, *SE* = .07, *p* < .001) and higher incomes (*b* = .50, *SE* = .08, *p* < .001). Therefore, H6 was supported.

Regarding H7, the moderation effect of residential area between HEC and individuals’ Twitter adoption was found to be non-significant (*b* = .14, *SE* = .13, *p* = .30). Therefore, H7 was supported.

## Discussion

This study integrated household- and individual-level data through a multilevel modeling approach, thereby considering the influence of household factors, specifically household economic capital (HEC), on an individual’s Twitter adoption behavior. The findings revealed both direct and indirect significant associations between HEC and Twitter adoption among household members, mediated by factors such as activity variety and self-reported efficacy in mobile technology use. Additionally, the study identified that the educational levels of household members and their individual income levels served as moderating factors in the relationship between HEC and Twitter adoption.

First, this study’s findings showed a correlation between a household’s economic capital and its members’ likelihood of adopting Twitter. This aligns with Pew Research Center’s observations in the U.S. [33], suggesting that individuals from wealthier families are more inclined to use Twitter. The reason may be that Twitter and other forms of social networking sites are provided with the function of information seeking, networking expansion and social identity construction in virtual space, which are forms of online social capital [50, 51]. However, the benefits of online social capital are often not immediately apparent [52]. People from lower-income households may have fewer opportunities or intentions to perceive the relative advantage of online social capital and, consequently, are less likely to adopt it [53]. A household with greater economic capital, in contrast, typically possesses other forms of capital, e.g., cultural, social, intellectual et al., equipping their members with diverse resources to recognize and appreciate the value of innovation such as Twitter.

Meanwhile, this study discovered a positive association between HEC and activity variety on mobile technology, which subsequently affects an individual’s Twitter adoption. This means that economic factors influence not only the mobile technology adoption [17, 18], but also the range of activities conducted on these devices. People coming from higher-income households tend to exploit the multifaceted functionalities of mobile technology more comprehensively, such as not just browsing websites and taking photos, but also experimenting with an array of installed applications to manage their lives. In other words, there is a higher probability for people coming from wealthy families to have their everyday activities, including social activities, reappear on mobile devices. This comprehensive engagement with mobile technology, in turn, increases their propensity to become Twitter users.

Additionally, the results showed that people’s household financial situation influenced self-reported ease of use on mobile devices, which is in line with Rogers’s theory on diffusion of innovation. According to Rogers, the complexity of an innovation, as perceived by members of a social system, is inversely related to its rate of adoption [53]. Thus, higher income of household could reduce its members’ perceived complexity of activity on mobile technology, while increasing the incentives to use social networking sites. In a nutshell, the findings of this study indicated that a person’s household economic capital can be transformed into its members’ capacity on mobile devices, which can be represented by (a) the diversity of one’s activity on mobile technology, (b) the subjective perception on one’s ability to use mobile technology, and (c) the online behavior.

The study also uncovered that the influence of HEC on Twitter adoption is moderated by individual sociodemographic characteristics. Specifically, it was observed that the lower an individual’s income and educational level, the more pronounced the impact of household income on their social networking site usage. This phenomenon can be attributed to the fact that individuals with limited financial and intellectual resources are likely to derive greater benefits from the support and resources provided by their family. Consequently, even if an individual possesses a lower educational background and earns less, their family’s resources can facilitate access to social networking sites. Conversely, individuals with higher levels of education and income typically have access to a broader range of social support and communication networks, which can aid their engagement in online spaces. Hence, the relative influence of household factors on such individuals is diminished.

The limitations of this study should be noted. First, household economic capital is measured using an item of family gross income. Household economic capital, however, can be embodied in a variety of contexts, like household saving and investment. More aspects of economic capital can thus be taken into consideration in future studies. In addition, future studies could look into other aspects of household capital, e.g., cultural capital, to fully comprehend the household influence on a person’s behavior in cyberspace. Second, this study employed a dichotomous variable, whether participants use Twitter or not, to measure Twitter usage, rather than analyzing specific activities on Twitter in more detail. Future studies could assess the use of social networking sites from more aspects, like activity frequency, activity level and activity type etc. Third, this study focused solely on the usage of Twitter. The results might not be applicable to other social media platforms with different functions.

Prior studies on social media adoption often focus on factors at individual level, such as that related to demographic features [6, 21] and psychological motivations [22, 23]. This study theoretically enhances understandings of household contextual elements that influence individuals’ adoption and usage of Twitter and mobile technology, further extending the literature related to media adoption The interaction effects between household- and individual-level also provide insights for the inconsistency of previous findings. Practically, the findings of this study could assist business organizations in formulating effective marketing campaigns, and for social institutions in addressing the digital divide.
